# The role of alternative microbial resources in competition between *Daphnia* species

**DOI:** 10.1093/plankt/fbag020

**Published:** 2026-04-07

**Authors:** Irina Feniova, Tomasz Brzeziński, Andrew R Dzialowski, Anna Bednarska, Bartosz Kiersztyn, Varos G Petrosyan, Natalia Zilitinkevich, Piotr Dawidowicz

**Affiliations:** Institute of Ecology and Evolution, Russian Academy of Sciences, Leninsky Prospect 33, Moscow 119071, Russia; Department of Hydrobiology, Institute of Ecology, Faculty of Biology, University of Warsaw, Żwirki i Wigury 101, 02-089 Warszawa, Poland; Department of Biology, 213 Life Sciences East, Oklahoma State University, Stillwater, OK 74078, United States; Department of Hydrobiology, Institute of Ecology, Faculty of Biology, University of Warsaw, Żwirki i Wigury 101, 02-089 Warszawa, Poland; Department of Hydrobiology, Institute of Ecology, Faculty of Biology, University of Warsaw, Żwirki i Wigury 101, 02-089 Warszawa, Poland; Institute of Ecology and Evolution, Russian Academy of Sciences, Leninsky Prospect 33, Moscow 119071, Russia; Water Problems Institute, Russian Academy of Sciences, Gubkina 3, Moscow 117971, Russia; Department of Hydrobiology, Institute of Ecology, Faculty of Biology, University of Warsaw, Żwirki i Wigury 101, 02-089 Warszawa, Poland

**Keywords:** competition, bacteria, alga, food quality, population dynamics

## Abstract

We studied competitive interactions between *Daphnia longispina* and *D. magna* competing for a single algal resource of varying quality in treatments with low and enhanced amounts of bacteria. We proposed that the outcome of competition is influenced by the concentration of bacteria. We used three types of food including high-quality green alga *Chlamydomonas klinobasis*, phosphorus-poor *C. klinobasis* and the cyanobacterium *Synechococcus elongatus*, which is deficient in essential lipids. The medium in the low-bacteria experiment was replaced daily, while the medium in the enhanced-bacteria experiment was changed every other day, thus allowing for prolonged bacterial growth as determined by an additional short-term experiment. Furthermore, we found significantly higher abundances of both *Daphnia* species in monocultures with enhanced bacteria compared to those in the low-bacteria experiment, indicating that they were feeding on bacteria. In the experiment involving low bacterial densities, the dominance of both *Daphnia* species depended on the quality of the algae. However, *D. magna* was the superior competitor across all food quality treatments in the enhanced bacteria experiment. We conclude that enhanced bacteria are a significant factor that shapes competitive interactions in cladoceran communities.

## INTRODUCTION

Competition between cladoceran species is a central topic in plankton ecology (Gliwicz, 2003). A set of hypotheses and ideas has been suggested to predict the outcomes of competition between cladocerans including the size-efficiency hypothesis (Brooks and Dodson, 1965), the *r*-max hypothesis (Goulden *et al.,* 1978), the resource competition theory (Tilman, 1981, 1982) and the threshold food concentration hypothesis (Gliwicz, 2003). These hypotheses can be categorized into two distinct classifications: universal hypotheses, which make predictions based on a metric of competitive ability, and conditional hypotheses in which competitive abilities shift in response to specific conditions. The second group of hypotheses assumes that there are trade-offs between traits that may increase a species’ competitive ability in one set of environmental conditions but decrease its relative fitness in another. For example, large daphnids have a higher efficiency of resource exploitation than small species (Gliwicz, 2003) but they can lose their competitive advantage in an environment that is deficient in eicosapentaenoic acid (EPA) because large species have a higher EPA demand (Sikora *et al.,* 2016). In this respect, we posited that bacteria might also facilitate the growth of one species over the other in competition due to more efficient consumption of bacteria.

Predation has often been cited as a factor mitigating competition within zooplankton communities and is ultimately more important than exploitative competition in shaping their structure (Dodson, 1974; Dawidowicz and Pijanowska, 1984; Arnott and Vanni, 1993). However, in some habitats, such as fishless ponds or oligotrophic lakes, zooplankton is limited by food (Tessier and Goulden, 1982; Dawidowicz and Pijanowska, 1984; Gliwicz, 1985; Müller-Navarra and Lampert, 1996; Guisande *et al.,* 2000). Since lakes provide a variety of food sources (e.g. algae, bacteria, protozoans and detritus), trophic niche differentiation between species may determine the composition of the zooplankton community. The more selective feeding of a species, the greater its niche segregation. Daphnids are filter feeders, and they show less selective feeding than copepods (DeMott, 1986). For this reason, cladocerans have a higher degree of food niche overlap compared to copepods (Guisande *et al.,* 2003).

Despite the fact that all *Daphnia* species have similar modes of feeding, they can differ due to their unique features. For example, the filtering appendages of *Daphnia magna* Straus have very small mesh sizes (Geller and Müller, 1981). As a result, *D. magna* can acquire bacteria more efficiently than representatives of the *D. longispina* complex. Still, *D. longispina* O. F. Müller can graze on bacterioplankton. For example, in Lake Paione Superiore (Northern Italy), the contribution of bacteria to the diet of *D. longispina* ranged from 1% to 42%, with the highest percentages observed during periods characterized by a high proportion of juveniles (Riccardi, 2002). The *D. longispina* population consumed between 0.04% and 1.8% of bacterioplankton and between 0.3% and 17.6% of phytoplankton standing stocks per hour (Riccardi, 2002). Based on experimental data, the above author concluded that bacteria provide a supplementary food item, exploited largely when the supply of phytoplankton declines.

Another important factor in shaping zooplankton communities is food quality. Shortage of polyunsaturated fatty acids (PUFAs) or phosphorus (P) can affect a species’ fitness, thus altering competitive interactions (Iwabuchi and Urabe, 2012; Sikora *et al.,* 2016; Feniova *et al.,* 2024, 2025). The species that are less sensitive to shortages of essential substances gain an advantage in competition. For example, Sikora *et al.* (2016) found that large-bodied *Daphnia* had higher EPA thresholds than small-bodied cladocerans, which implied that large-bodied species should be weaker competitors when EPA is limiting *Daphnia* growth and reproduction. Similarly, P deficiency is important in exploitative competition, and limitation in this essential element in food may be pivotal in shaping species structure in cladoceran communities (Iwabuchi and Urabe, 2012; Feniova *et al.,* 2025).

Heterotrophic bacteria can contribute considerably to the nutrition of *Daphnia* species in nature (Perga *et al.,* 2006; Taipale *et al.,* 2008, 2009). It was shown that marine bacteria produce PUFAs including EPA and docosahexaenoic acid (DHA) (Okuyama *et al.,* 2007; Gladyshev *et al.,* 2013). While less is known about freshwater bacteria, Dailey *et al.* (2015) isolated four strains of EPA-producing free-living freshwater bacteria. In addition, there is evidence that sterol synthesis occurs in freshwater bacteria (Wei *et al.,* 2016). Bacteria also provide *Daphnia* with P in environments where algae are P-poor (Hessen and Andersen, 1990; Iwabuchi and Urabe, 2010). Hessen *et al.* (1990) reported that in a humic lake, bacterial carbon accounted for 11–42% of the body carbon in cladocerans, including *Daphnia*, while phytoplankton contributed only 6–19%. Moreover, Iwabuchi and Urabe (2010) highlighted that bacteria can function as an alternative resource shaping competitive outcomes among daphnids, particularly under P and carbon limitation. These results underscore the central role that bacteria can play in the diet of cladocerans.

The species with a lower threshold food concentration (food concentration at which birth rate equals death rate) should be a superior competitor (Tilman, 1982). That is the case when two cladoceran species compete for one limiting resource such as algae. However, when an alternative resource such as bacteria is available, competitive advantage for the main resource may be outweighed by the effectiveness in acquiring the second resource. We tested this hypothesis with two species of *Daphnia* that varied in their body size and competitive ability. Adults of the studied clone of *D. magna* were much larger than those of the *D. longispina* clone (Feniova *et al.,* 2024). According to Gliwicz (1990), the threshold food concentration of larger *D. magna* is lower than that of *D. longispina*. We expected that the outcome of competition between *D. longispina* and *D. magna* would differ under different amounts of bacteria. We conducted competition experiments with three types of food quality including high-quality (P-rich) green alga *Chlamydomonas klinobasis,* P-poor *C. klinobasis* and low-quality cyanobacterium *Synechococcus elongates* that lacks essential lipids. We had two sets of competition experiments with each of the three food quality types. In the first set, bacterial development was minimized through daily exchange of water, which was preliminarily filtered to remove bacteria. In the second set, bacteria were allowed to develop through less frequent medium exchange that occurred every other day. We regarded bacteria as an additional source of food, alongside algae, and suspected that their influence would affect the outcome of competition. In an additional trial, we studied the growth of bacteria under similar experimental conditions, but without *Daphnia*. This was to show that the experiment where we changed the medium every other day resulted in bacterial enrichment, compared to the experiment in which we replaced the medium daily.

## MATERIAL AND METHODS

### Description of the material and medium preparation

We used *D. magna* (clone DMN—Novy Vrbensky Rybnik, Czech Republic) and *D. longispina* (clone GB01—Großer Binnensee, Germany) in our experiments. A single clone of each species was maintained at the Department of Hydrobiology, University of Warsaw. *Daphnia magna* and *D. longispina* are closely related freshwater planktonic cladoceran species. Both are filter feeders and do not show selective feeding or the ability to discriminate between food particles based on chemosensation (Gliwicz, 1990). They often co-occur in nature where they directly compete for food (Ranta, 1979; Hanski and Ranta, 1983; Slusarczyk *et al.,* 2012). Both species co-existed in the lake from which the *D. longispina* clone was isolated (Grosser Binnensee, Germany) to use in our experiment.

Body length, dry body mass and filtration rates at first reproduction are presented for the studied clones of *D. longispina* and *D. magna* based on measurements of 15 individuals (Table I). Adults of *D. magna* were approximately twice as large (body length) and eight times heavier (dry body weight) than those of *D. longispina* (Table I). Filtering rates were estimated according to Burns (1969). The size of the species determines the principal traits of ecology and physiology, thus resulting in different life strategies and competitive ability (Hart and Bychek, 2011). The variations in size in females between the studied species are greater than the range of clonal variation within each species (Slusarczyk *et al.,* 2012). In addition, the efficiency of bacteria consumption depends on species identity (e.g. Geller and Müller, 1981). Thus, we do not think that the results of our competition experiments would be different if we tested multiple clones of each species.

**Table I:** Means (±SD) of body length, dry body mass and filtration rates at first reproduction of D. longispina and D. magna (N = 15)

Species	Body length (mm)	Dry body mass (μg)	Filtering rate (mL/(ind^*^h)
*D. magna*	1.54 ± 0.04	28.45 ± 1.97	0.56 ± 0,03
*D. longispina*	2.67 ± 0.09	227.19 ± 35.69	2.10 ± 0.16

We used three types of food quality: (i) high-quality green alga *C. klinobasis* with C:P ≈ 100 (HQ); (ii) P-poor *C. klinobasis* with C:P ≈ 800 (PP); and (iii) cyanobacteria *S. elongatus* (CYANO). *Chlamydomonas klinobasis* (strain #56) was collected from the Limnological Institute, University of Constanz. *Chlamydomonas klinobasis* does not contain eicosapentaenoic acid (EPA), which is necessary for daphnids, but this alga contains alpha-linolenic acid ALA (18:3ω3), which is a precursor for synthesis of the long-chain omega-3 including EPA (von Elert and Fink, 2018). *Synechococcus elongatus* (SAG89.79) was cultivated in the Department of Hydrobiology, University of Warsaw. Since *S. elongates* lacks PUFAs and sterols (Martin-Creuzburg *et al.,* 2008) without which the growth and reproduction of *Daphnia* is impossible, the cyanobacteria treatment was supplemented with 5% P-rich *C. klinobasis.*

The cyanobacterium was grown in a chemostat with Wright's Chu#10 (WC) medium (Guillard, 1975) at 20 ± 1°C, light intensity 56 ± 1 μmol × m^−2^ × s^−1^ and harvested at the stationary phase of growth. *Chlamydomonas klinobasis* was cultivated in batch cultures (0.5 L Erlenmeyer flask filled with 0.2 L of medium) in full WC medium (HQ) and WC medium depleted with phosphorus (PP, 0.1 × of nominal phosphorous content). Batch cultures were cultivated on a shaker (PSU-20i, Biosan, Poland) at 100 rpm, in a thermostatic chamber (ST35, Pol-Eko Aparatura, Poland) at 20 ± 2°C and light intensity 35 ± 1 μmol × m^−2^ × s^−1^. Batch cultures of *C. klinobasis* were harvested after 4 days (P-rich medium) or 8 days (P-depleted medium) of incubation. Harvested algae and cyanobacteria were stored in the fridge until use (8°C).

The C content in algae was measured using a FLASH 2000 Elemental Analyzer (ThermoFisher Scientific Inc., USA), from which carbon-extinction equations were determined. The C content of the algal suspensions was obtained by reference to photometric light extinction at 800 nm using a Perkin-Elmer spectrophotometer (Perkin-Elmer, USA). The P content was determined by the conventional molybdenum blue method using a Skalar San++ analyzer.

To prepare the medium for *Daphnia,* we diluted filtered lake water with demineralized water in the proportion 1:2 (lake water: demineralized water) to reduce the concentration of inorganic salts in the medium. Lake water was collected from the eutrophic Lake Szczęśliwickie located in Park Szczęśliwicki (district Ochota, Warsaw, Poland). Concentration of inorganic phosphorus in summer to autumn varied from 0.0194 to 0.0003–0.0007 mg P-PO_4_^3−^ L^−1^. N-NH_4_^+^ varied in the range of 0.0003 mg N L^−1^ –0.0194 mg N L^−1^ (Siuda, 2014). We filtered lake water through a Sartoban R capsule filter (pore size 0.2 μm) to remove bacteria. Simultaneously with water exchange, we cleaned and dried experimental containers. Since the experiments were not performed in sterile conditions, microorganisms including bacteria could develop in the medium. We assumed that replacing the medium every other day (~48 hours) would result in a significantly higher amount of both suspended bacteria and biofilms on the walls than if the medium were replaced daily (~24 hours) with fresh bacteria-free medium.

### Experimental design

**Fig. 1:**
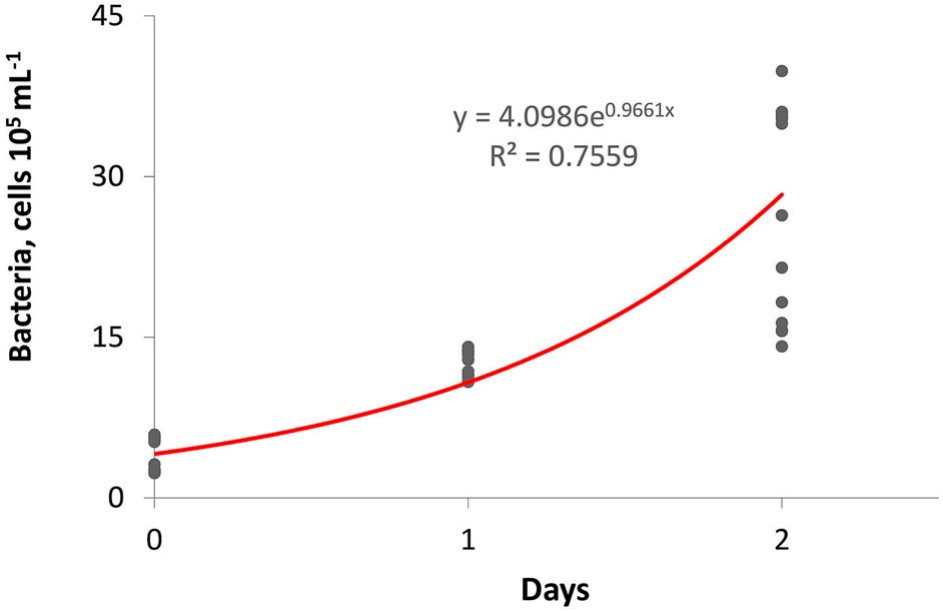
Regression relationships between concentration of bacteria and the time of medium exposure.

We conducted a short-term experiment to verify that more bacteria developed in the medium changed every other day compared to the daily changed medium. We measured the abundance of bacteria in a *Daphnia*-free medium that was supplied daily with equal proportions of *C. klinobasis* and *S. elongatus* with a daily food supply of 0.18 mg C L^−1^. Bacteria samples were collected after 24 and 48 hours of exposure in the medium in 400 mL jars at 24°C with a photoperiod of 16 L:8D, which represented conditions in the competition experiments. Bacteria were stained with DAPI (4,6-diamidino-2-phenylindole) (DAPI BN), and the total number of cells was counted on 0.2 μm black polycarbonate membrane filters (Millipore) using an epifluorescence microscope (Porter and Feig, 1980). DAPI at a final concentration of 1 μg/mL was used for 10 minutes at 24°C. We applied a computerized imaging analysis system to count the bacteria with a Nikon E450 epifluorescence microscope, a Nikon DXM 1200F digital camera and NIS-Elements software (Nikon). Bacteria were counted from digital images of 10 random fields for each membrane filter (50–400 bacteria per field, image area: 5510 μm^2^, UV-2A Nikon fluorescence filter—Ex. 330–380 nm: DM. 400 nm, Em. 420 nm). Based on our assumption and the results of the experiment with bacteria (Fig. 1), we determined that changing the water every day (A = algae/cyanobacteria experiment) had less bacteria compared to when we changed the water every other day (B&A = bacteria and algae/cyanobacteria experiment). In the latter case, we expected that competition could occur for both algae/or cyanobacteria and bacteria.

Competition experiments were performed in 400 mL glass jars kept in a water bath at a temperature of 24°C ± 0.1°C regulated by Zefir immersion thermostats (Adarex, Poland) and under a summer photoperiod (16 L:8D). Experimental vessels were exposed to low-light conditions (1.25 μmol photons/m^2^/s or 23 lx) to inhibit the growth of algae added to the medium. We had similar treatments for each of the two sets of experiments (A and B&A) containing different combinations of each food type with monocultures (single species) and mixed (two species) cultures of *D. longispina* and *D. magna.* In total, we had 18 treatments (2 water-renewal experiments × 3 types of food quality × 3 *Daphnia* treatments, two monocultures and one mixed culture), each treatment replicated in triplicate. In each jar, we placed *D. longispina* and *D. magna* (20: 5 ind.) neonates born within 12 hours from females kept prior to the experiments in the conditions corresponding to the respective experimental treatments. The initial number of *D. longispina* neonates was higher than that of *D. magna* to create a similar starting biomass of each species. We assumed that the initial number of the neonates would not influence the carrying capacity (stable period of abundance) of the environment when competition occurred. We used the same number of *Daphnia* to start both the monoculture and the mixed culture treatments. The daphnids were fed with food at a concentration of 0.09 mg C L^−1^ twice a day at a 12-hour interval. We selected discrete feeding of *Daphnia* in our competition experiment because preliminary testing of a flow-through system showed an accumulation of bacteria due to bacterial contamination of the tubes and chambers, which we could not change often. In the current design of discrete feeding, it was possible that some food remained 12 hours after feeding in the B&A experiment, but it was so small that the difference in algal food supply during the feeding between the two sets of experiments was negligible and had no effect on demographic parameters. The degree of decline of algae/cyanobacteria concentration in the experimental treatments for 12 hours after feeding during the equilibrium state of the cultures in the B&A experiment was larger than in the A experiment, and the remaining algae/cyanobacteria ranged from 0.03 to 0.02 mg C L^−1^. We do not believe that this had a significant effect on reproduction and duration of juvenile development. According to our previous life-table experiments, significant differences of demographic parameters between species were found between 0.09 and 0.18 C L^−1^ (Feniova *et al.,* 2025).

Every sixth day, we counted the number of individuals in each jar under a microscope (Nikon SMZ1000, Nikon Corporation, Tokyo, Japan) and measured chlorophyll concentration using a PhytoPam fluorometer (Heinz Waltz GmbH, Germany). The experiments lasted for 36 days including 24 days of stable growth of daphnid abundances and chlorophyll concentrations.

### Statistical analysis

A regression analysis between medium exposure time and bacterial abundance was conducted to justify the division of the experiments into two types (A and B&A). For the competition experiments, we used three- and five-way general linear models with repeated-measures analysis of variance (GLM RM ANOVA) to assess the effects of experimental set (A vs. B&A), food quality (FQ: HQ, PP and CYANO), culture type (two monocultures vs. mixed culture) and sampling date on the abundances of *D. magna* and *D. longispina* during the experiments from Day 12 to Day 36, when food concentration was maintained at the minimum level. In all GLM RM ANOVAs, Jar and Date were treated as random factors, whereas all other factors were regarded as fixed factors. The initial phase of increasing abundance (from the start of the experiment to Day 12) was not analyzed because food was not limiting during this period. Tukey’s *post hoc* tests (*P* < 0.05) were applied to identify significant pairwise differences in abundances among treatments detected by the GLM RM ANOVA. Abundance data were log-transformed prior to analysis to meet the assumptions of residual normality and homoscedasticity. All statistical analyses and figure preparation were performed in R version 3.3 (R Core Team, 2025) and NCSS version 7.

The list of the abbreviated terms is given at the end of the Supplementary material.

## RESULTS

Bacteria grew exponentially in the medium without *Daphnia* so that there were more bacteria after 48 hours compared to 24 hours (Fig. 1). Bacteria concentrations increased after 48 hours by an average of more than six times, reaching 2.7× 10^6^ cells mL^−1^ compared to 4.1 × 10^5^ cells mL^−1^ at the start.

It should be noted that both species of *Daphnia* did not reproduce in the CYANO treatment in the A experiment because *D. longispina* died before the first reproduction after 14–15 days of the experiment, while *D. magna* persisted until the end of the experiment without producing any eggs and zero population growth. However, they did reproduce in the CYANO treatment in the B&A experiment. For this reason, the dynamics of the abundances of *Daphnia* in the CYANO treatment in the A experiment were not considered.

**Fig. 2:**
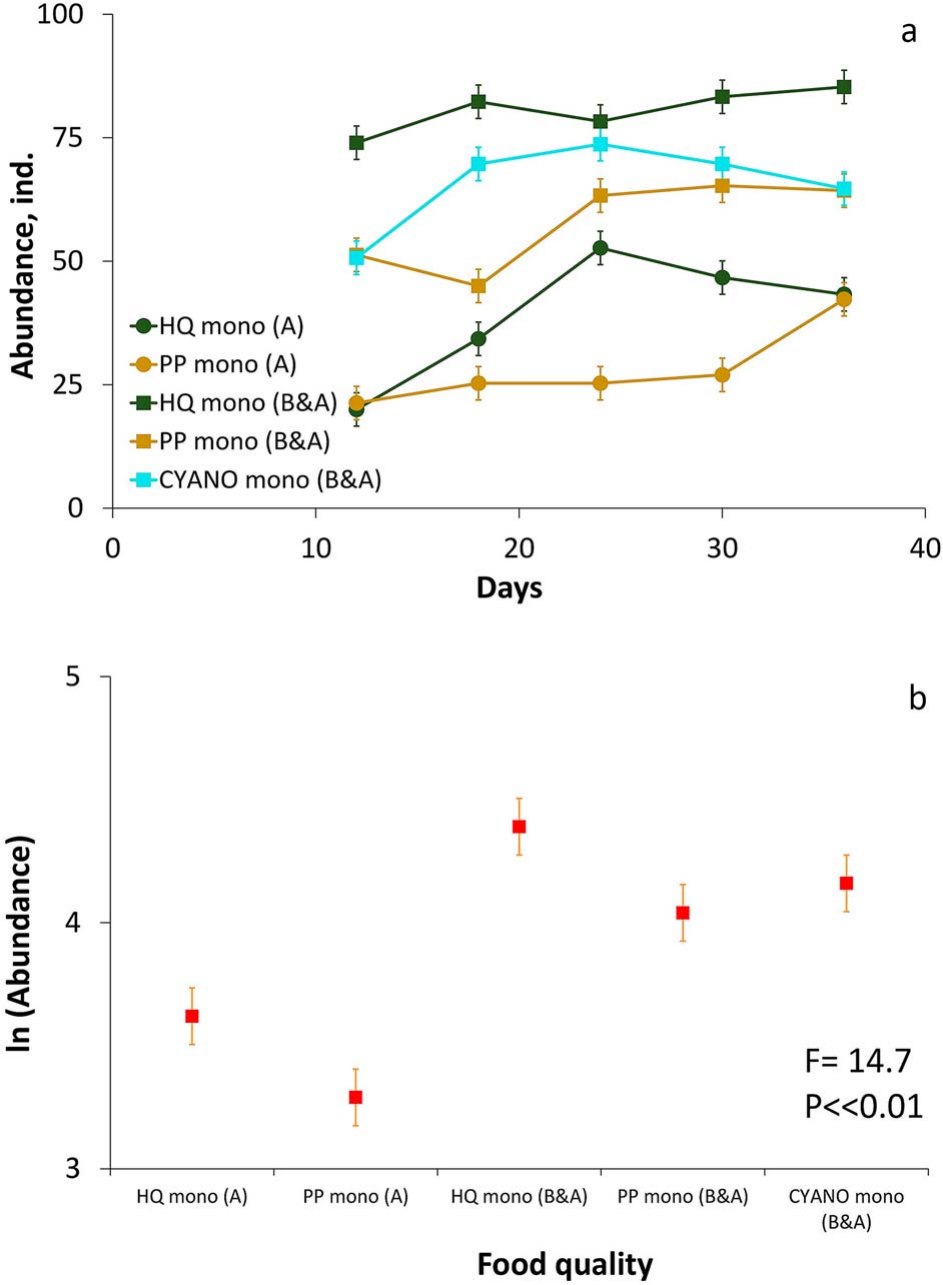
Dynamics of the abundance **(a)** and means **(b)** of the abundances in *D. longispina* in different food quality treatments in monocultures in A and B&A experiments. Error bars (a and b panels) denote 95% of Tukey’s honestly significant difference (HSD) intervals. HQ, high-quality algae treatment; PP-P, poor algae treatment; CYANO, cyanobacteria treatment with addition of 5% of high-quality algae; A, low-bacteria experiment; B&A, high-bacteria experiment.

**Fig. 3:**
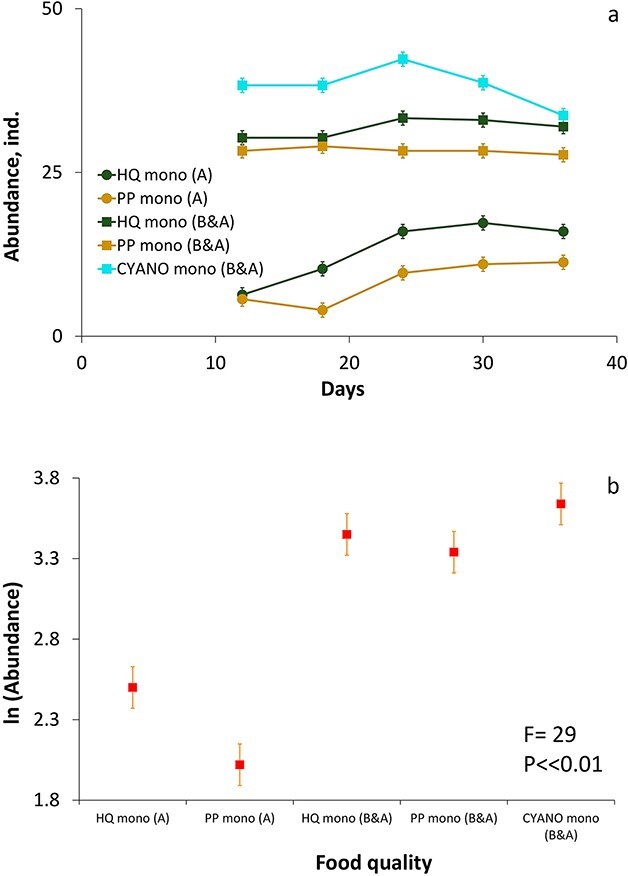
Dynamics of the abundance **(a)** and means **(b)** of the abundances in *D. magna* in different food quality treatments in monocultures in A and B&A experiments. Error bars (a and b panels) denote 95% of Tukey’s HSD intervals. The designations of the legends are described in Fig. 2.

In the monocultures, the abundances of both *Daphnia* species were higher in the B&A experiment compared to the A experiment across all food quality treatments (GLM RM ANOVA, Figs 2 and 3, Figs S1 and S2, Table S1). Food quality also influenced the abundances of both species (Tables S1–S3). The abundances of *D. longispina* during the equilibrium period were significantly greater in the HQ treatment compared to the PP and CYANO treatments in both sets of the experiment (Fig. 2b, Fig. S1). The abundances of *D. magna* were also significantly different in the HQ treatment compared to the PP treatment in the A experiment (Fig. 3b, Fig. S2). However, in the B&A experiment, its abundances in monocultures in the HQ treatment did not differ significantly from the PP and CYANO treatments (Fig. 3b, Fig. S2). Therefore, both *Daphnia* species responded significantly to food quality based on mean abundances, but more pronounced effects were observed between the A and B&A experiments that showed the positive influence of an enhanced amount of bacteria on the abundance of daphnids in monoculture.

**Fig. 4:**
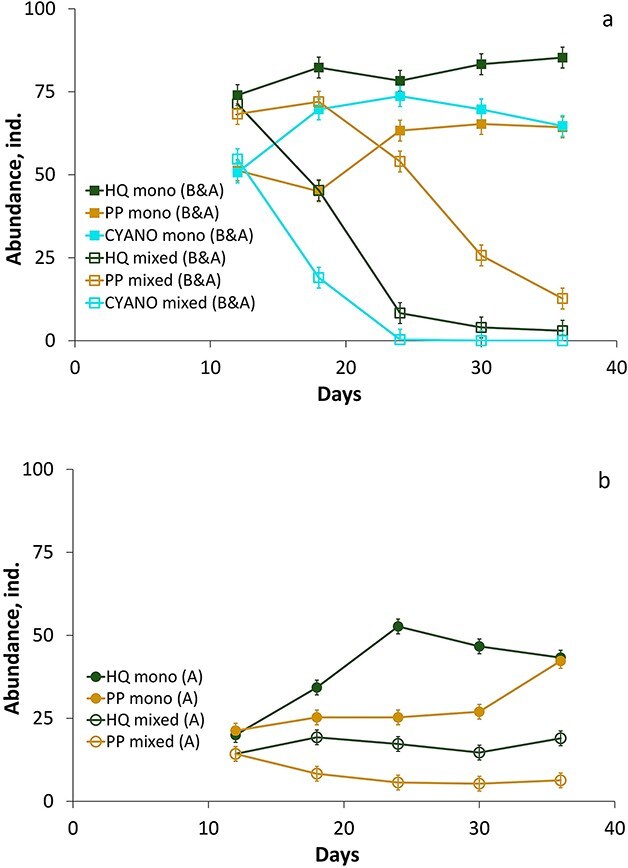
Dynamics of the abundance of *D. longispina* in different food quality treatments in mono- and mixed cultures in B&A **(a)** and A **(b)** experiments. Error bars (a and b panels) denote 95% of Tukey’s HSD intervals. The designations of the legends are described in Fig. 2.

**Fig. 5:**
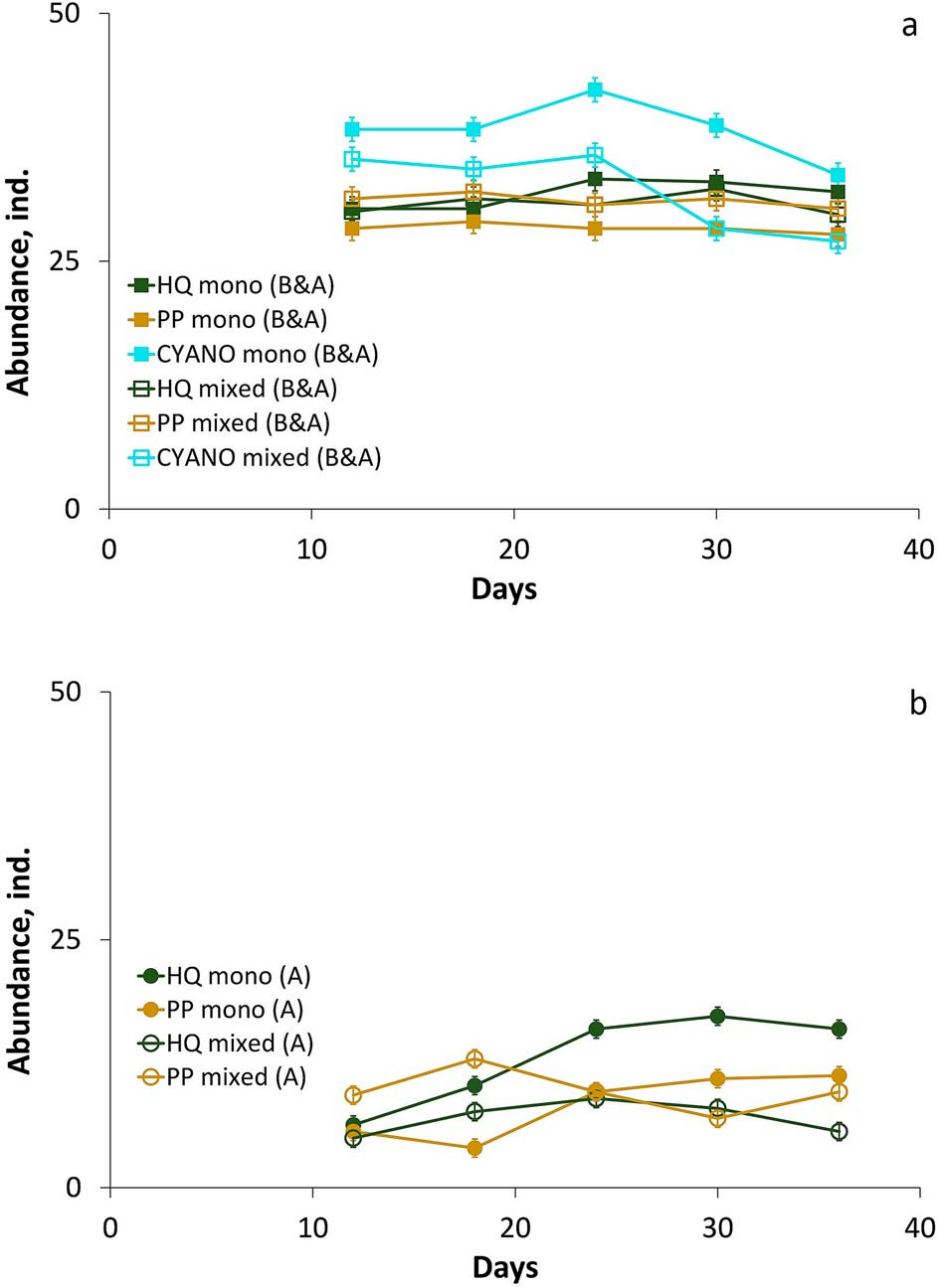
Dynamics of the abundance of *D. magna* in different food quality treatments in mono- and mixed cultures in B&A **(a)** and A **(b)** experiments. Error bars (a and b panels) denote 95% of Tukey’s HSD intervals. The designations of the legends are described in Fig. 2.

In the mixed cultures, we could not separate interspecies competition and food quality effects. However, we found that the dynamics of the two species were different. In particular, *D. longispina* decreased in all the treatments to either be fully excluded in the CYANO treatment or close to zero in the HQ and PP treatments in the B&A experiment (Fig. 4a, Fig. S3). However, abundances of *D. longispina* in the A experiment were sustainable in the HQ treatment, while they declined in the PP treatment approaching zero (Fig. 4b, Fig. S4). *Daphnia magna,* on the contrary, sustained its abundance in the mixed cultures of the B&A experiment at levels similar to the monocultures in the HQ and PP treatments, and there were no significant differences between mono- and mixed cultures in the HQ and PP treatments (Fig. 5a, Fig. S5). However, in the CYANO treatment, the abundances of *D. magna* in the monocultures were higher than in the mixed cultures (Fig. 5a, Fig. S5). In contrast, in the A experiment, there were significant differences between mono- and mixed cultures of *D. magna* in the HQ treatment for most of the stable period and its abundance approached zero (Fig. 5b, Fig. S6). Therefore, competition outcomes were different in the A and B&A experiments. In particular, *D. longispina* was excluded in the HQ treatment in the B&A experiment while it successfully persisted in this food quality treatment and even dominated over *D. magna* in the A experiment*.* In contrast, *D. magna* was strongly suppressed in the A experiment (HQ treatment) but was superior in the B&A experiment in all the treatments.

Algae/cyanobacteria dynamics are provided in Supplementary Material to show the periods when food concentration was sustained at the minimum levels and the extent of food concentration decline (Figs S7 and S8). The degree of decline of algae/cyanobacteria concentration in the experimental treatments after 12 hours during the equilibrium state of the cultures is presented in Table S4. In the B&A experiment, the decline of food supply for 12 hours ranged from 3- to 4.3-fold, while in the A experiment varied in the range 1.9–2.9-fold because the total abundance of the individuals was less in the A experiment.

## DISCUSSION

Both algae and bacteria can be important resources for zooplankton. Hessen *et al.* (1990) showed that the total epilimnetic zooplankton community, on average, removed 9 μg of algal C and 10 μg of bacterial C L^−1^ d^-l^. Bacterial carbon accounted for 11–42% of zooplankton body carbon, while phytoplankton accounted for only 6–19%. We expected that cladocerans consumed both algae and bacteria in our experiments because they are filter feeders (DeMott, 1986). In turn, we anticipated that the experiment where bacteria developed for a longer period of time would facilitate the growth of the species that consumed the additional resource more efficiently and ultimately alter the outcome of competition for a single food resource. Indeed, bacterial concentrations increased exponentially over time, with more rapid growth occurring during the second day. This pattern suggests that conditions became increasingly favorable for bacterial proliferation as the experiment progressed. In addition, the number of bacteria that developed in our experimental jars after 48 hours was typical of eutrophic lakes in the temperate zone (3.9–5.2 × 10^6^ cell mL^−1^, Kiersztyn *et al.,* 2019), while the abundance of bacteria after water filtration corresponded to oligotrophic conditions (0.2–1.4 × 10^6^ cells mL^−1^, Kopylov and Kosolapov, 2007). Therefore, the concentrations of bacteria that developed in our experiments can be considered as ecologically relevant.

The significantly higher population abundances of both *Daphnia* species in the monocultures in the B&A experiment, regardless of food quality treatment, suggest that bacteria growing in the medium were an important additional food source. In support, *Daphnia* did not reproduce in the CYANO treatment in the A experiment, where bacteria were present in low numbers. Furthermore, *D. magna* grew poorly in the monocultures on a diet of phosphorus-deficient algae (PP) in the low-bacteria experiment (A) while in the B&A experiment with enhanced bacteria, *D. magna* had similar abundances in the P-poor, CYANO and HQ food treatments. Hence, the bacteria in this experiment were likely not only an additional source of carbon for *Daphnia* but also improved the overall food quality. Still, in the case of *D. longispina,* its abundance was lower in the treatments with P-poor food relative to high food quality treatment, both in the low- (A) and high-bacteria (B&A) experiments. These observations suggest that *D. magna* benefited more than *D. longispina* from the enhanced bacteria in low-quality food treatments (PP and CYANO). As we indicated previously, the remaining food concentration before each feeding was too small to influence demographic parameters of *Daphnia*.

There is evidence in the literature that algal P shortage in experimental conditions can be mitigated due to nutrient recycling by *Daphnia* (Urabe *et al.,* 2002). However, algae did not develop in our experiment during the analyzed period of the equilibrium state, as occurred in Urabe *et al.* (2002). The discrete feeding at the 12-hour interval facilitated the rapid removal of newly added algae from the experiment. Besides, we observed effects of P shortage on both *Daphnia* species in the A experiment and on *D. longispina* in the B&A experiment. Therefore, we can suggest that the increased growth of *D. magna* in the B&A experiment was due to its efficient consumption of bacteria and not an increase in algal food quality. Consistent with this interpretation, bacteria are known to mitigate P limitation in algal resources for cladocerans (Hessen and Andersen, 1990; Iwabuchi and Urabe, 2010).

Furthermore, interactions between *D. magna* and *D. longispina* in the mixed cultures were different in the two experiments. In the B&A experiment, *D. magna* was a superior competitor in all the food quality treatments, while in the A experiment, *D. longispina* dominated over *D. magna* in the high-quality treatment but not the P-poor treatment. Thus, algal quality may influence the outcome of competition between *Daphnia* in an experiment where algae were mostly present, but it may be less important in an experiment with enhanced bacteria. This finding is consistent with Pace *et al.* (1983) showing that the ability to consume bacteria can affect competitive interactions, potentially even resulting in the dominance of a more efficiently grazing bacterivore.

In our experiments, the presence of bacteria favored *D. magna* more than *D. longispina*. This is consistent with results reported in the literature. Specifically, when comparing grazing rates of *Daphnia*, Callieri *et al.* (2006) showed that the grazing rate of *D. magna* reached 6.4 × 10^6^ bacterial cells ind.^−1^ h^−1^ while the maximum grazing rate of *D. longispina* reached 3.5 × 10^6^ bacterial cells ind.^−1^ h^−1^. While bacteria usually constituted a small fraction of the diet of *D. longispina* relative to algae, the share of bacteria in the diet increased up to 55—73% when phytoplankton biomass declined (Kankaala, 1988). Bacteria can also become the dominant food source when algal resources are limited (Porter, 1984; Kankaala, 1988; Riccardi, 2002). The threshold phytoplankton concentration at which *D. magna* switched from feeding on algae to bacteria was reported at ~ 0.05 mg C L^−1^ (Siehoff *et al.,* 2009). In our experiments, food concentration declined ~3-fold as compared to the initial maximum 0.09 mg C L^−1^. Therefore, it is likely that when the algal concentration decreased at least 2-fold, *Daphnia* mainly competed for bacteria in the enhanced bacteria experiment. In addition to the bacteria in the water column, the slower exchange of water may have also resulted in increases in periphyton in the jars. Research shows that *D. magna* can use periphyton as an alternative food source when concentrations of green algae declined below 0.05 mg C L^−1^ (Horton *et al.,* 1979; Jones and Waldron, 2003; Siehoff *et al.,* 2009). This ability to switch between food sources may give *D. magna* a competitive advantage over *D. longispina*, which does not utilize periphyton as a food source. In support, Gliwicz (1990) indicated that the ability of *D. magna* to graze on periphytic bacteria provided a competitive advantage due to facilitation of its growth at very low algal concentrations. More periphyton accumulates on the bottom and walls of the experimental vessels when the water is exchanged at longer intervals. Therefore, when the algal concentration decreases, *D. magna*, which feeds on bacterioplankton more efficiently and would potentially graze on periphyton, gains an advantage in competition with *D. longispina* under conditions of an enhanced amount of these alternative resources. Additional research is needed to determine if periphyton also influenced competition under these experimental conditions.

Bacteria are not only a source of carbon as they are also rich in phosphorus and have low C:P and N:P ratios. Bacterial assemblages from lakes exhibit P content up to 3% of their dry mass (Godwin and Cotner, 2015) and can be an essential source of P for some zooplankton species (Hessen and Andersen, 1990; Iwabuchi and Urabe, 2010). In addition, bacteria are able to synthesize long-chain PUFAs (Bell and Tocher, 2009; Gladyshev *et al.,* 2013) including, in rare cases, synthesis of EPA and DHA by free-living freshwater bacteria (Dailey *et al.,* 2015). For this reason, bacteria could enhance food quality of the diet, if algae quality is poor due to low P-content or deficiency of PUFAs such as in the case of CYANO. These studies are consistent with the results of our experiments.

The present study supports previous arguments that bacteria may be a key food resource influencing the outcome of competitive interactions (Pace *et al.,* 1983; Iwabuchi and Urabe, 2010). In particular, our experiments have shown that *D. longispina* had a lower threshold food concentration (Feniova *et al.,* 2025), and was competitively superior over *D. magna* in the high food quality and low bacteria conditions. However, *D. magna* can competitively suppress *D. longispina* in the same algae quality treatment but with enhanced bacteria likely due to better exploitation of bacteria.

## CONCLUSIONS

In summary, when algae or cyanobacteria are in limited supply, bacteria may become a key food source for filter-feeding cladocerans, compensating for quantitative or qualitative deficiencies in their diet. Moreover, bacteria can influence the competitive interactions between cladoceran species, sometimes even altering the outcome of competition compared to the competitive interactions for a single food resource. In some cases, the efficiency with which a cladoceran species acquires bacteria may be a more important determinant of its competitive ability than the threshold food concentration established in the laboratory using a single food resource. Since we found that the algal quality affected the outcome of the competition only in the experiment with the single algal resource but not at the enhanced bacterial concentration created by less frequent water exchange, it can be concluded that bacteria can mitigate the effect of the low quality of the algal food. Therefore, when competition for algal food in filter-feeding zooplankton communities is strong, bacteria can be regarded as a significant factor in shaping their structure.

## Supplementary Material

04_02_2026_supplementary_fbag020

## Data Availability

Data will be made available on request.

## Notes

Corresponding editor: Lars-Anders Hansson
