# Supplementary material for: The role of alternative microbial resources in competition between Daphnia species
Source: J Plankton Res. 2026 Apr 7;48(3):fbag020. doi: 10.1093/plankt/fbag020 (PMC13064842; doi:10.1093/plankt/fbag020)
Supplement: 04_02_2026_supplementary_fbag020 [file 04_02_2026_supplementary_fbag020.docx]

**Supplementary material**


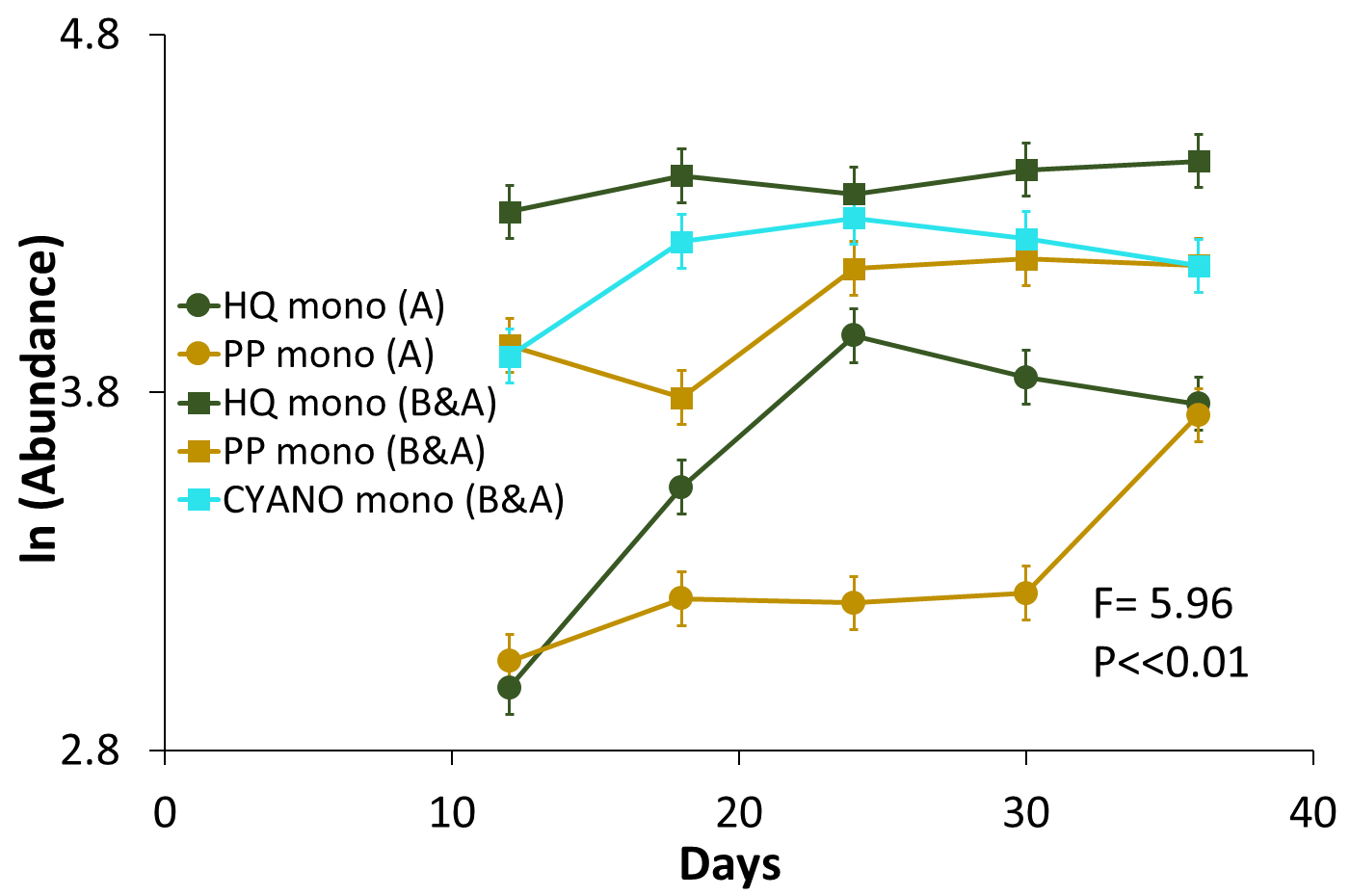


Figure S1. Dynamics of the abundance of *D. longispina* in different food quality treatments in monocultures in the A and B&A experiments. Error bars denote 95% of Tukey’s HSD intervals. High quality algae treatment – HQ, P-poor algae treatment – PP, cyanobacteria treatment with addition of 5% of high quality algae – CYANO, A – low-bacteria experiment, B&A – high-bacteria experiment. Y-axis is logarithmic.


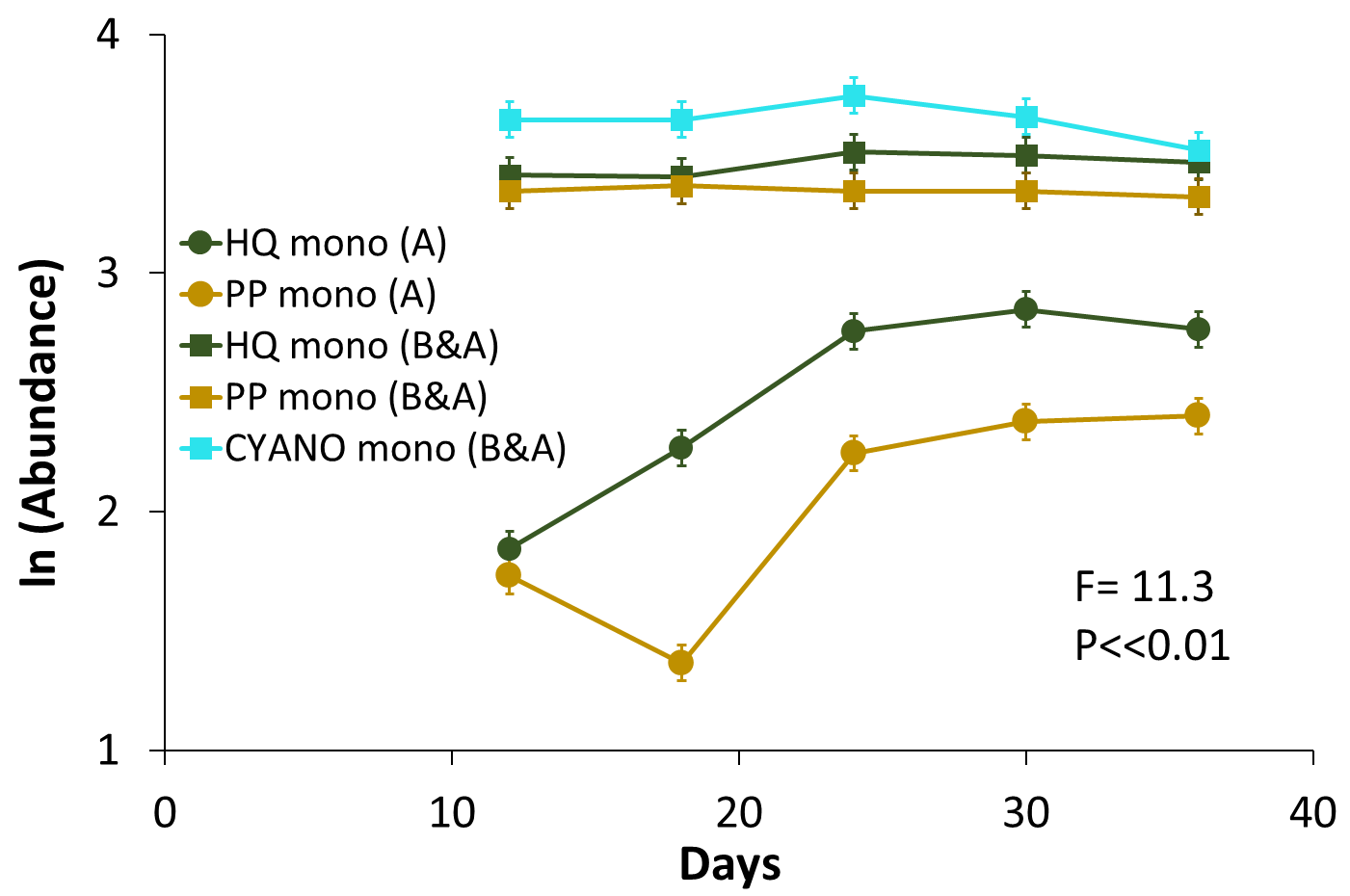


Figure S2. Dynamics of the abundance of *D. magna* in different food quality treatments in monocultures in the A and B&A experiments. Error bars denote 95% of Tukey’s HSD intervals. The designations of the legends are described in Fig. S1. Y-axis is logarithmic.


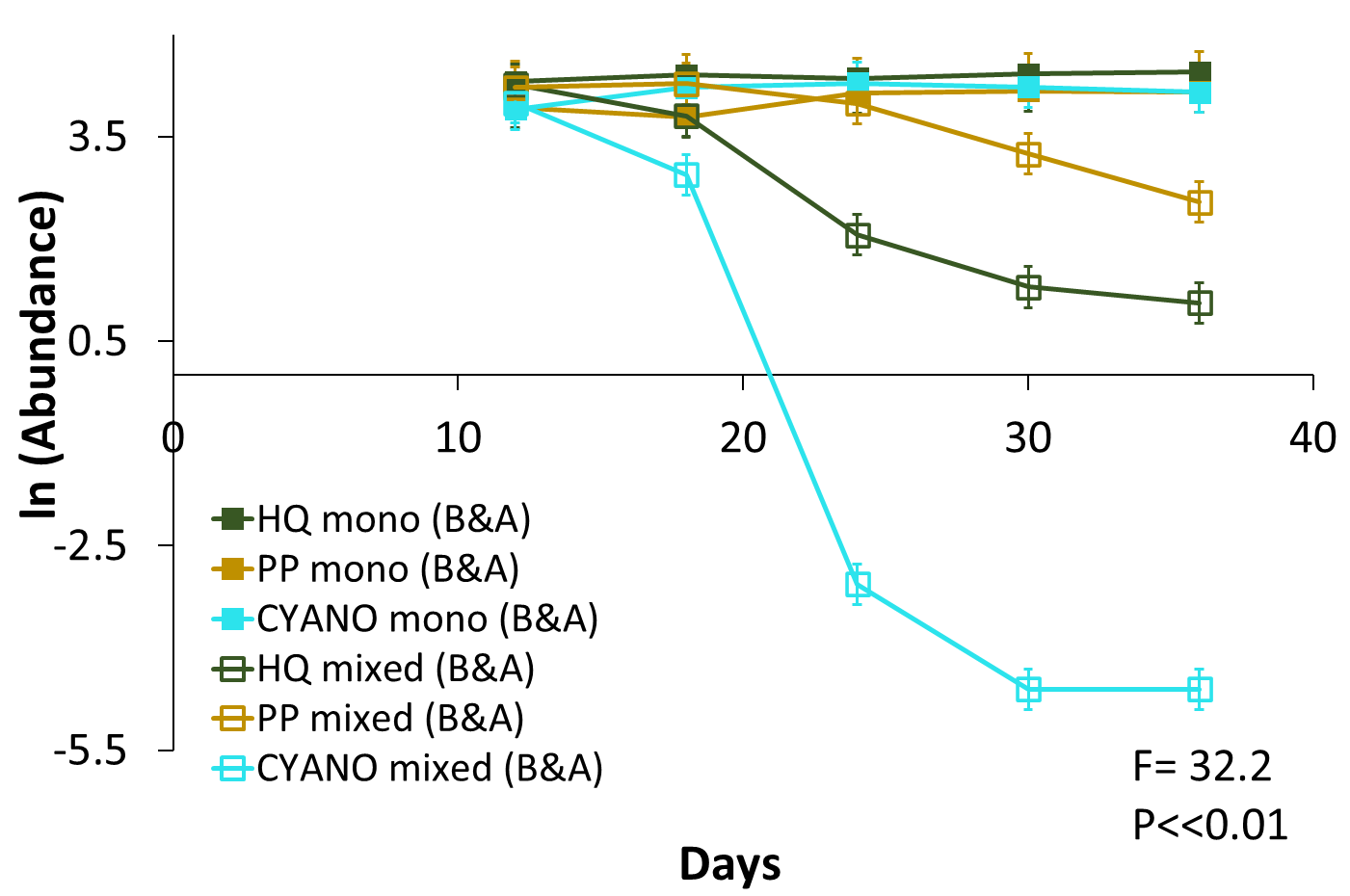


Figure S3. Dynamics of the abundance of *D. longispina* in different food quality treatments in mono- and mixed cultures in the B&A experiment. Error bars denote 95% of Tukey’s HSD intervals. The designations of the legends are described in Fig. S1. Y-axis is logarithmic.


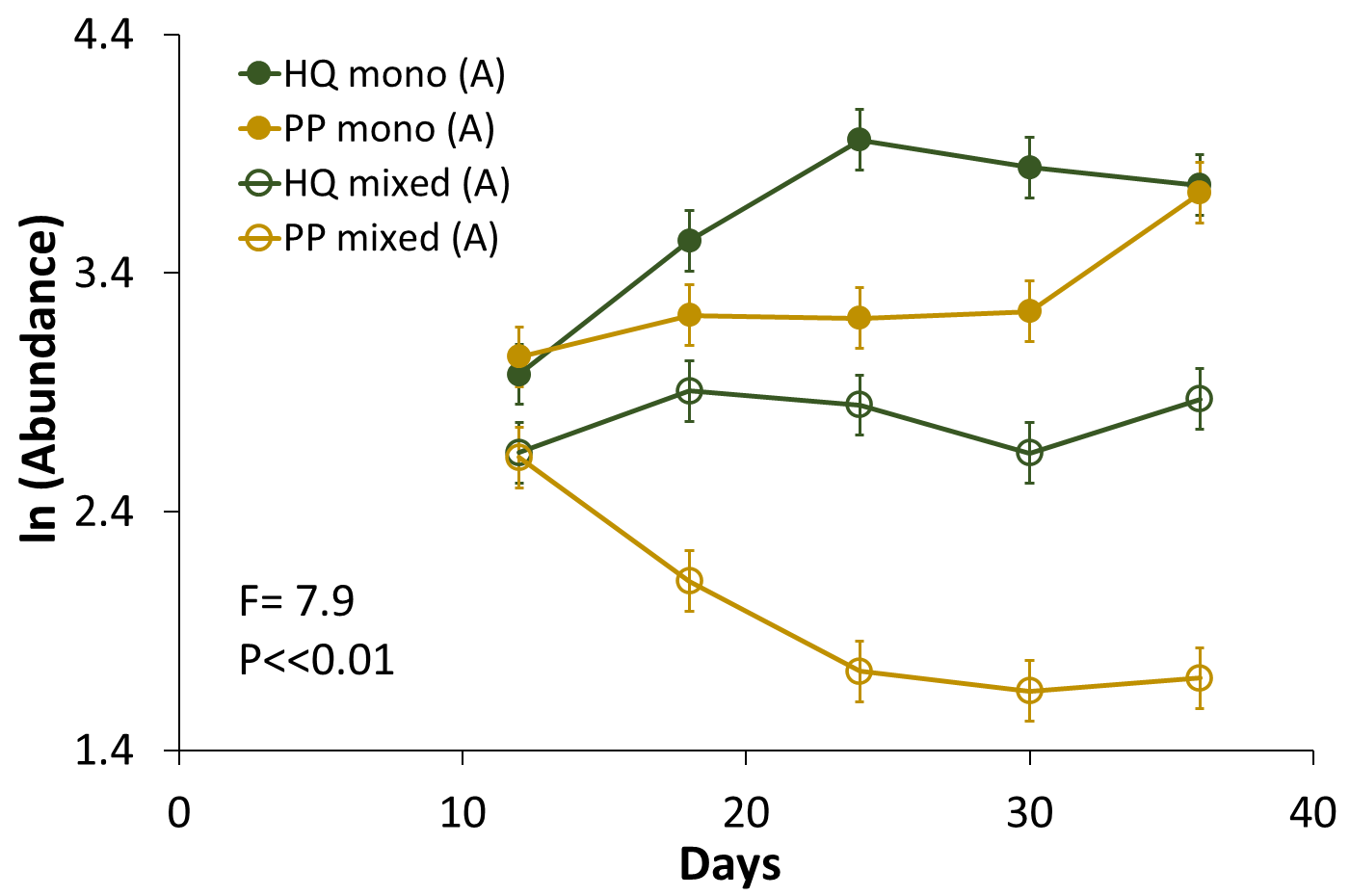


Figure S4. Dynamics of the abundance of *D. longispina* in different food quality treatments in mono- and mixed cultures in the A experiment. Error bars denote 95% of Tukey’s HSD intervals. The designations of the legends are described in Fig. S1. Y-axis is logarithmic.


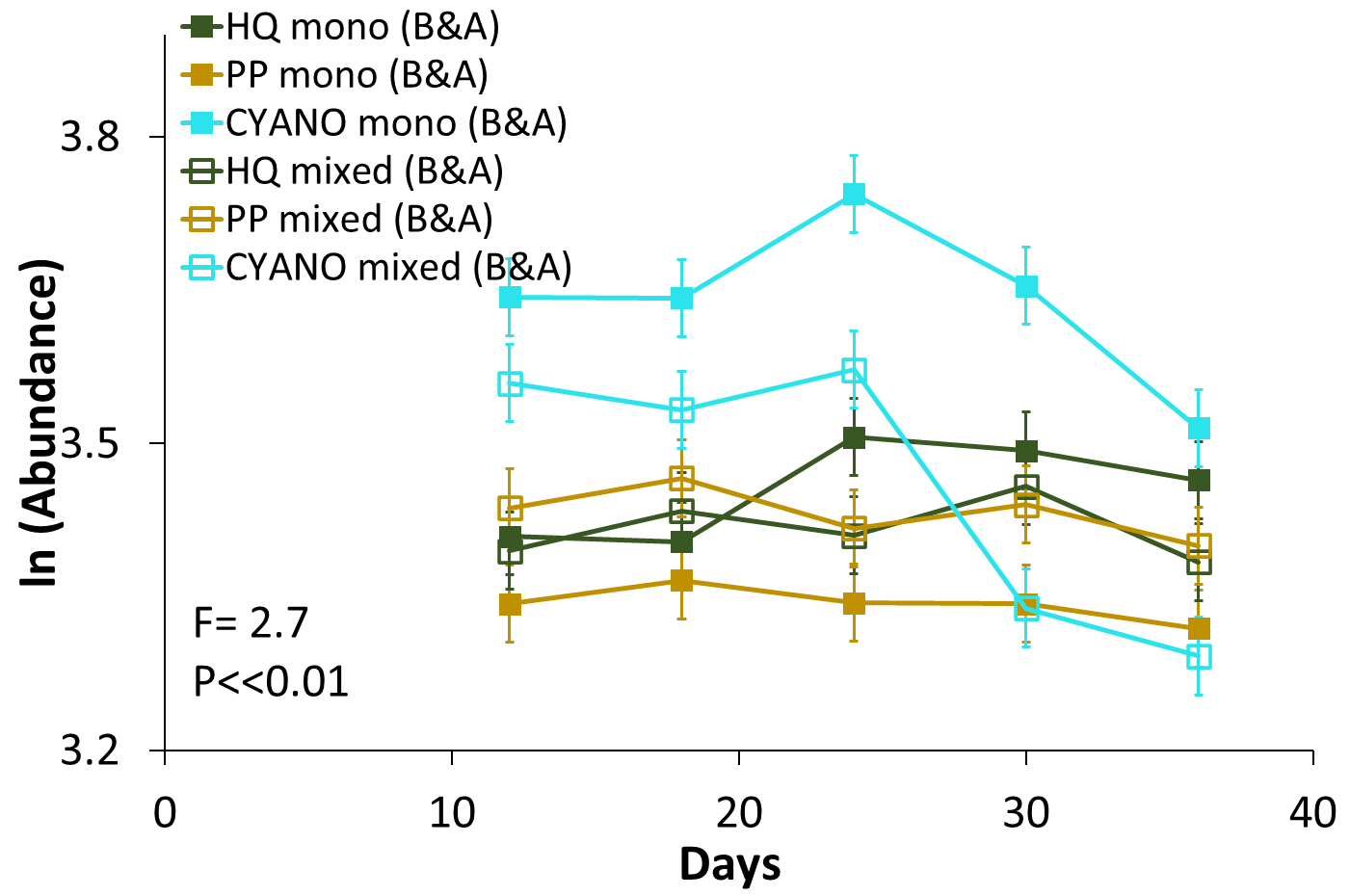


Figure S5. Dynamics of the abundance of *D. magna* in different food quality treatments in mono- and mixed cultures in the B&A experiment. Error bars denote 95% of Tukey’s HSD intervals. The designations of the legends are described in Fig. S1. Y-axis is logarithmic.


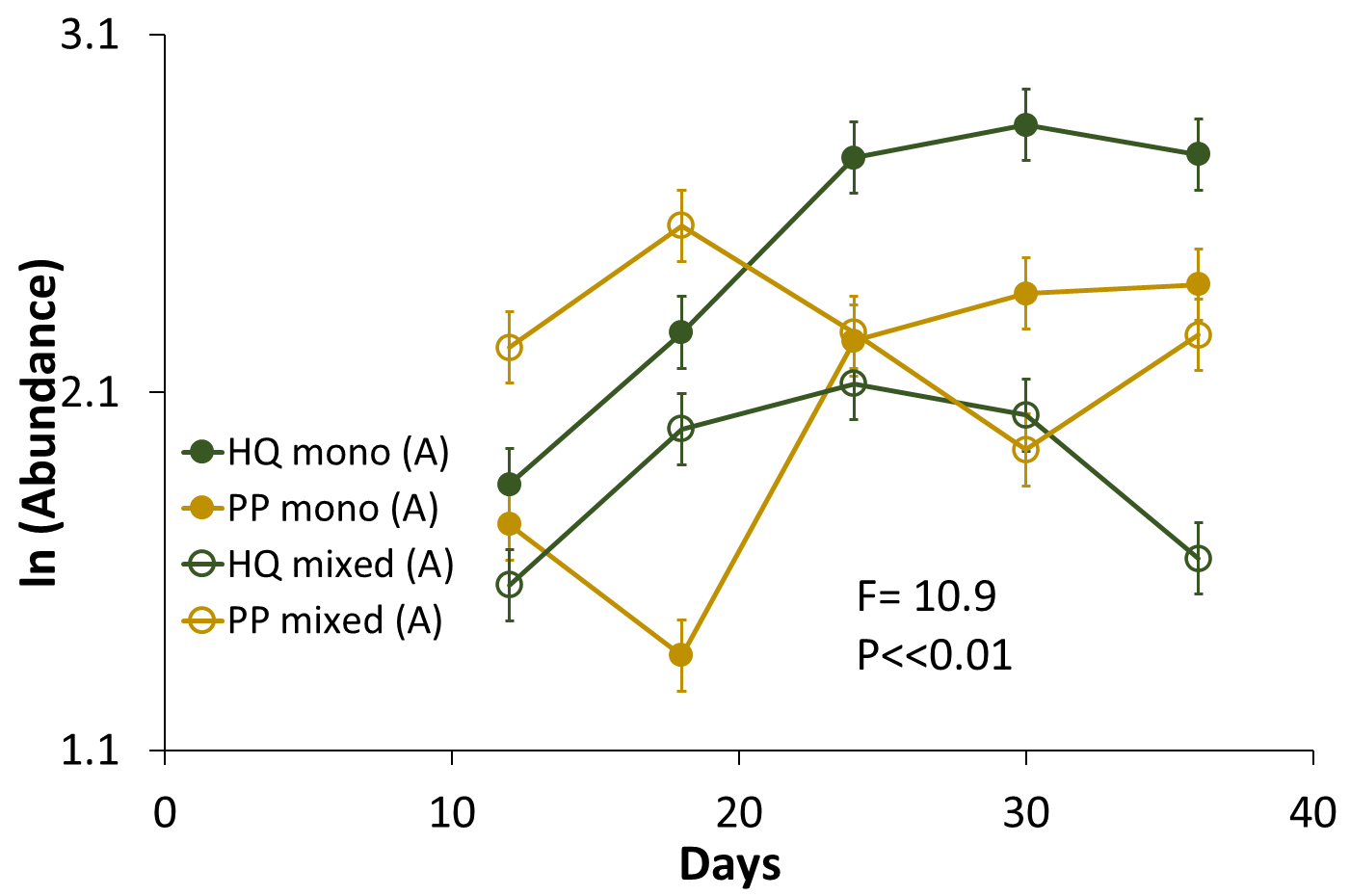


Figure S6. Dynamics of the abundance of *D. magna* in different food quality treatments in mono- and mixed cultures in the A experiment. Error bars denote 95% of Tukey’s HSD intervals. The designations of the legends are described in Fig. S1. Y-axis is logarithmic.


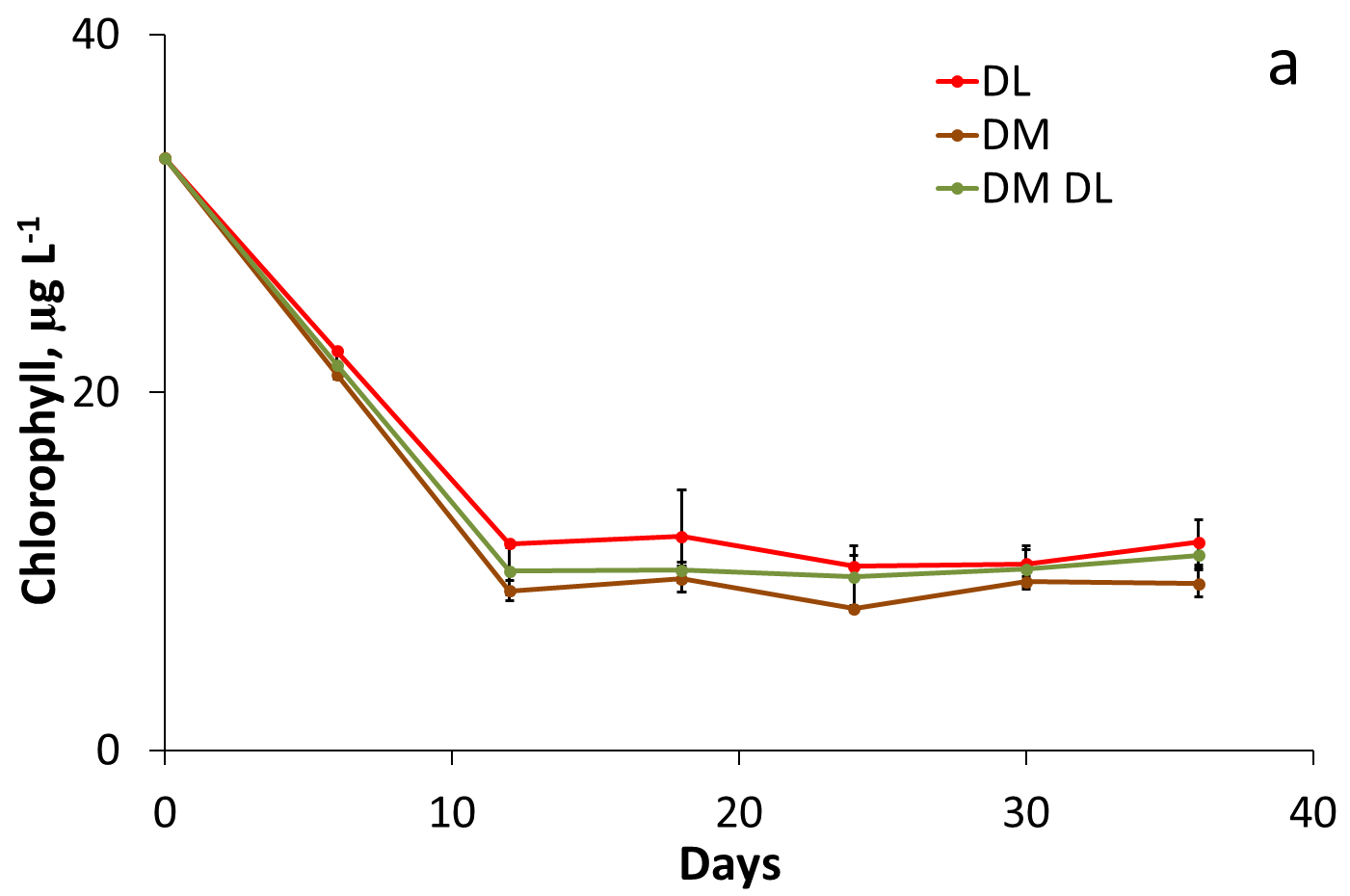


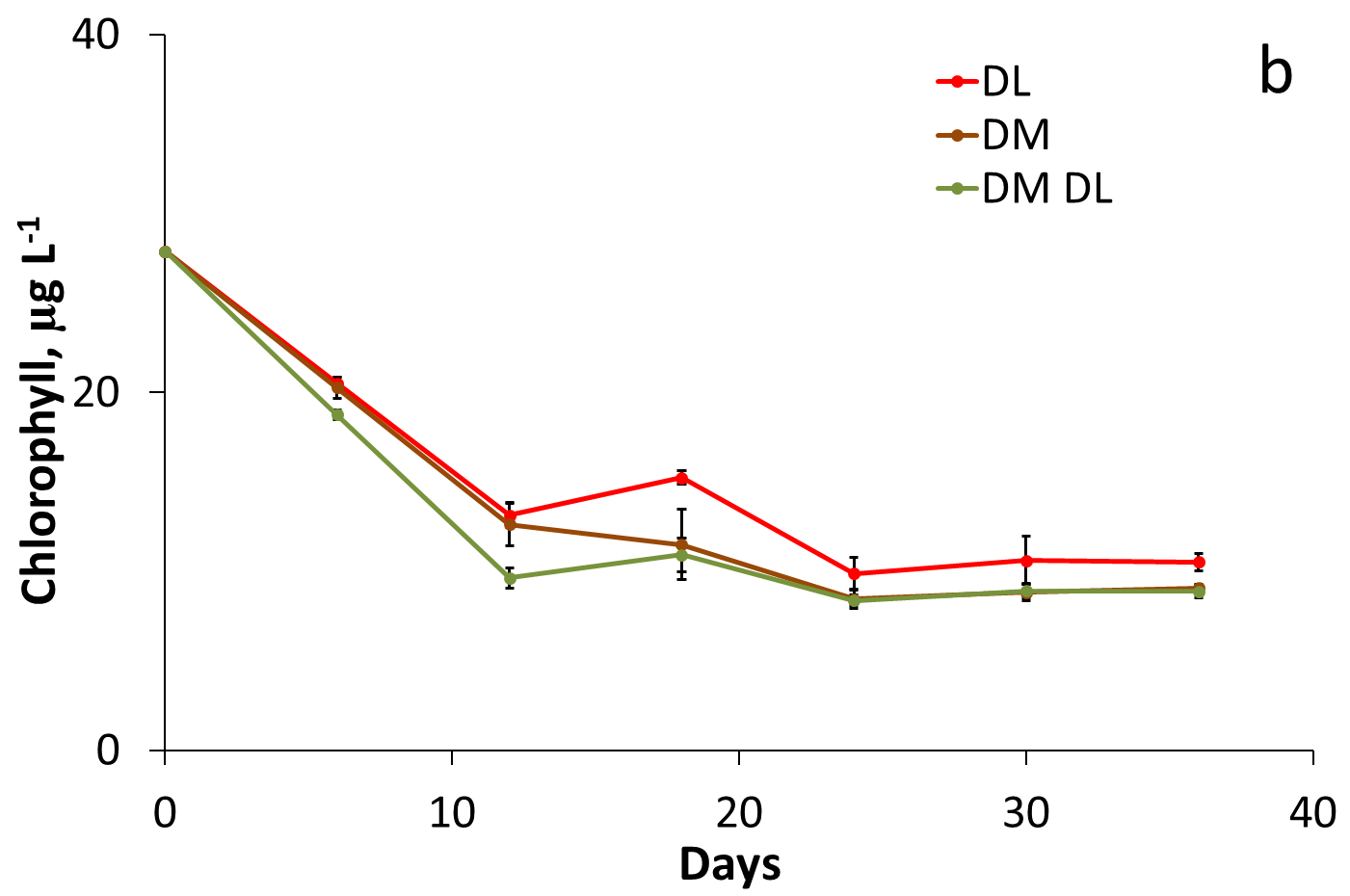


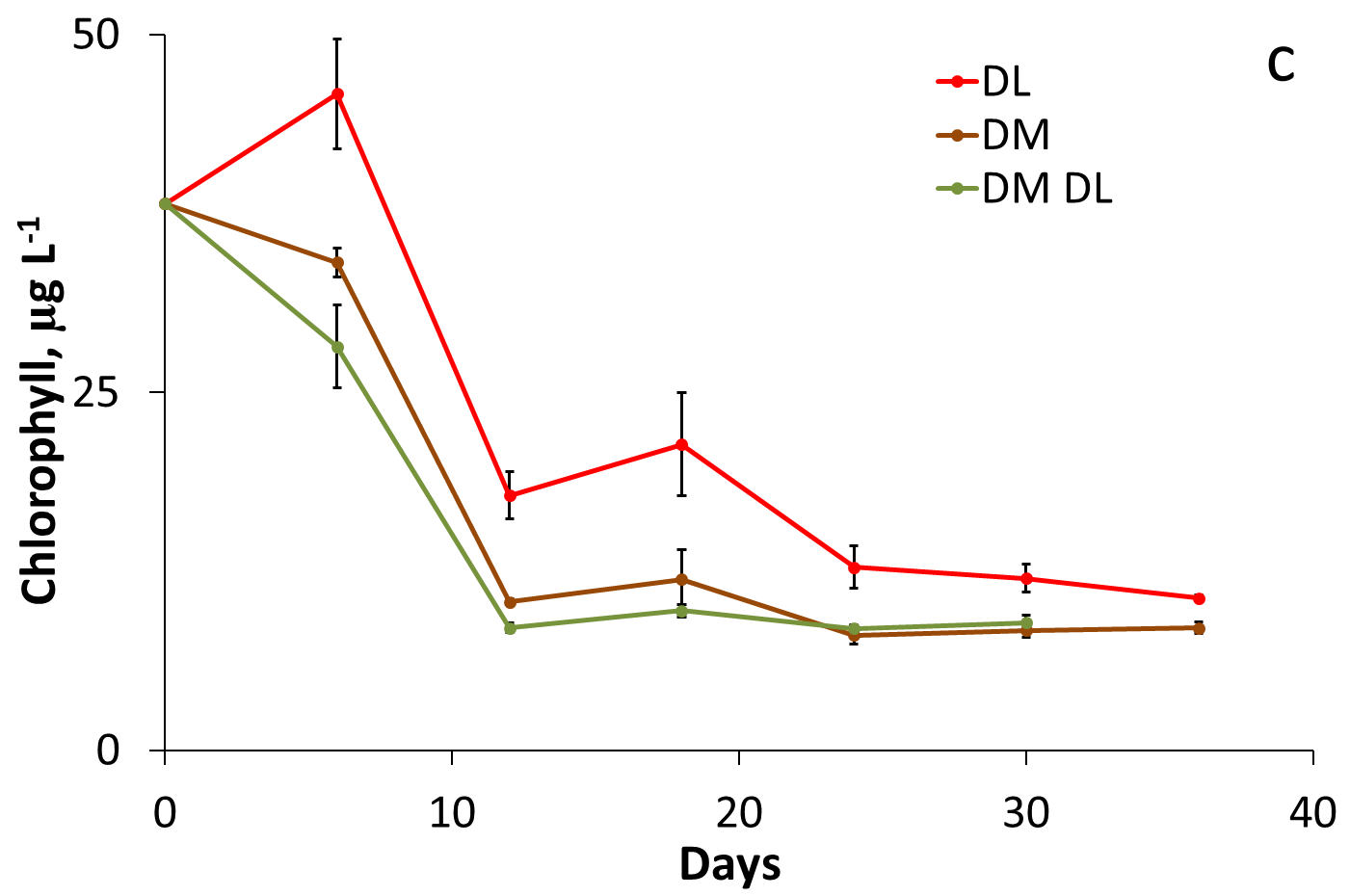


Figure S7. Dynamics of chlorophyll concentration in HQ (A), PP(B) and CYANO (C) in mono and mixed treatments with *D. longispina* and *D. magna* in B&A experiment. DL *–* *D. longispina*, DM *–* *D. magna.*


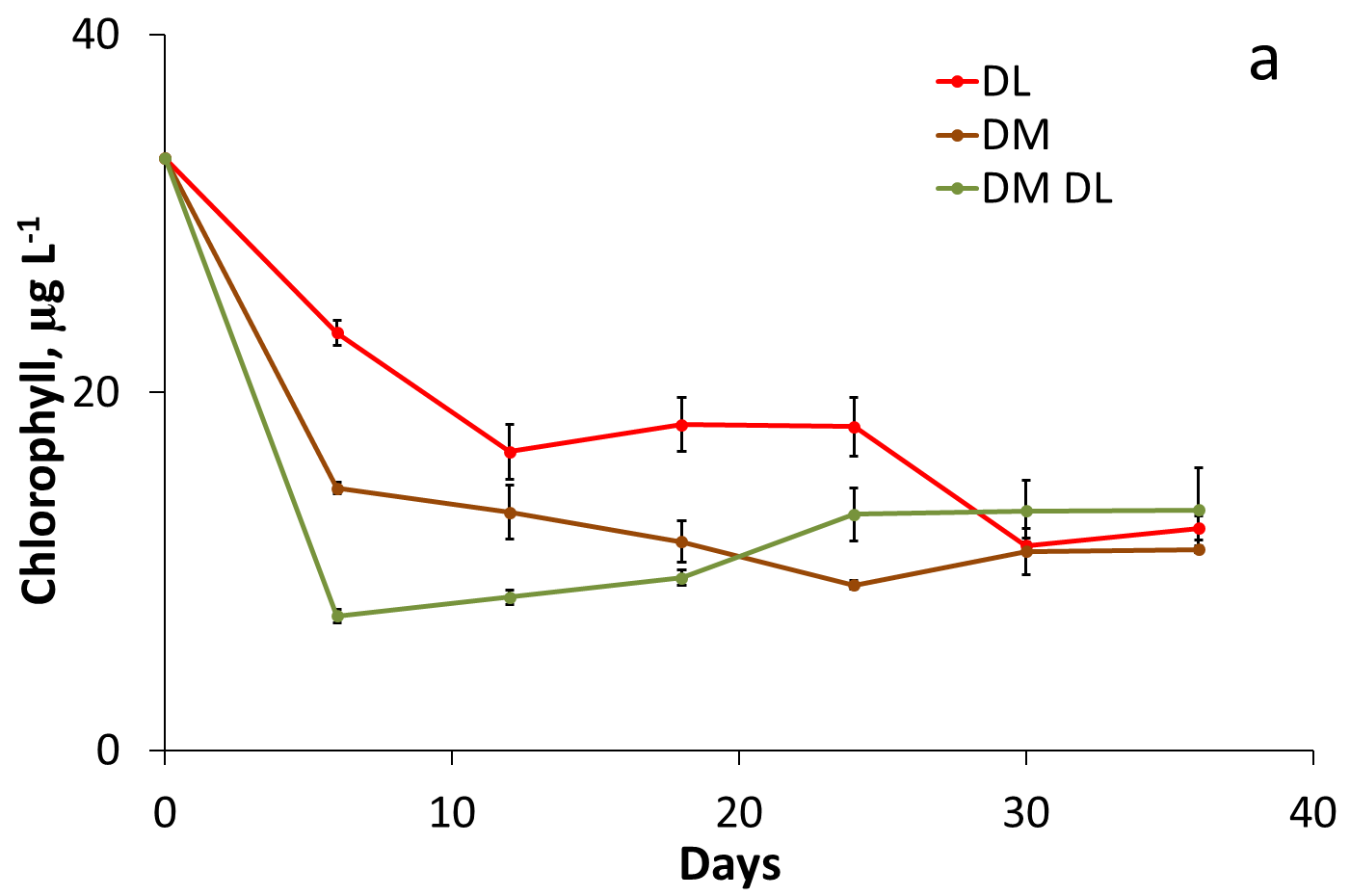


HP- algae


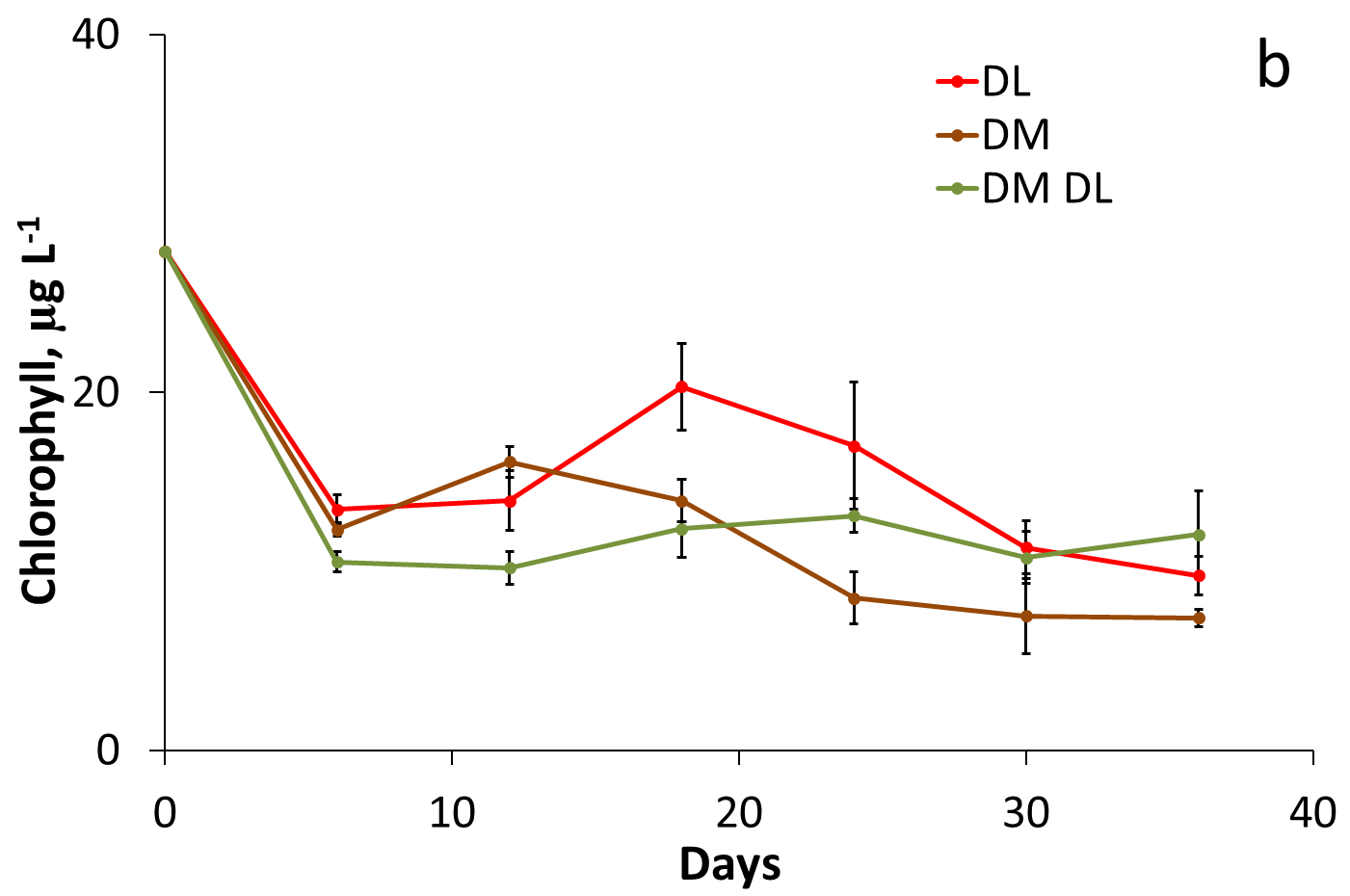


Figure S8. Dynamics of chlorophyll concentration in HQ (A) and PP(B) in mono and mixed treatments with *D. longispina* and *D. magna* in A experiment. DL *–* *D. longispina*, DM *–* *D. magna.*

Table S1. Results of two GLM RM ANOVA (1, 2) models with two random (Date, Jar) and three fixed factors—Type of_experiment (A, B&A), Food quality (HQ, PP, Cyano), and Culture (mono, mixed)—and interactions of fixed factors influencing the abundance of D. longispina (Dl) or *D. magna (*Dm), where the F-ratio is Fisher’s statistic and the P-value refers to the factor effects.

| Source | GLM RM ANOVA 1 | | GLM RM ANOVA 2 | |
| --- | --- | --- | --- | --- |
|  | Dl | | Dm | |
|  | F-Ratio | P-Value | F-Ratio | P-Value |
| Date | 5.81 | **<<0.01** | 2.24 | 0.07 |
| Jar | 0.02 | 0.97 | 0.96 | 0.39 |
| Type_of_experiment | 8.95 | **<<0.01** | 96.7 | **<<0.01** |
| Food quality | 44.1 | **<<0.01** | 7.6 | **<<0.01** |
| Culture | 102.6 | **<<0.01** | 120 | **<<0.01** |
| Type_of_experiment ×Food quality | 2.15 | 0.15 | 1.3 | 0.25 |
| Type_of_experiment × Culture | 0.01 | 0.9 | 3.58 | **0.06** |
| Food quality× Culture | 23.9 | **<<0.01** | 9.95 | **<<0.01** |

Table S2. Results of three GLM RM ANOVA models with two random (Date, Jar) and one fixed factors (food quality— HQ, PP, Cyano), and interaction of date and food quality influencing the abundance of D. longispina (Dl), where the F-ratio is Fisher’s statistic and the P-value refers to the factor effects.

| Source | GLM RM ANOVA 1 | | GLM RM ANOVA 2 | | GLM RM ANOVA 3 | |
| --- | --- | --- | --- | --- | --- | --- |
|  | DL, mono - Fig. S1 | | DL, B&A- Fig. S3 | | DL, A- Fig. S4 | |
|  | *F-Ratio* | *P-Value* | *F-Ratio* | *P-Value* | *F-Ratio* | *P-Value* |
| Date | 4.29 | **0.02** | 2.1 | 0.12 | 0.2 | 0.93 |
| Jar | 6.82 | **<<0.01** | 1.13 | 0.36 | 4.63 | **<<0.01** |
| Food quality | 14.69 | **<<0.01** | 7.4 | **<<0.01** | 14.9 | **<<0.01** |
| Date×Food quality | 5.96 | **<<0.01** | 32.2 | **<<0.01** | 7.9 | **<<0.01** |

Table S3. Results of three GLM RM ANOVA models with two random (Date, Jar) and one fixed factors (food quality- HQ, PP, Cyano), and interaction of date and food quality influencing the abundance of *D. magna* (Dm), where the F-ratio is Fisher’s statistic and the P-value refers to the factor effects.

| Source | GLM RM ANOVA 1 | | GLM RM ANOVA 2 | | GLM RM ANOVA 3 | |
| --- | --- | --- | --- | --- | --- | --- |
|  | Dm, mono- Fig. S2 | | Dm, B&A-Fig. S5 | | Dm, A-Fig. S6 | |
|  | *F-Ratio* | *P-Value* | *F-Ratio* | *P-Value* | *F-Ratio* | *P-Value* |
| Date | 2.4 | 0.09 | 2.3 | 0.10 | 1.7 | 0.22 |
| Jar | 4.6 | **<<0.01** | 12.6 | 0.09 | 9.8 | **<<0.01** |
| Food quality | 29.2 | **<<0.01** | 2.4 | 0.08 | 1.9 | 0.17 |
| Date×Food quality | 11.3 | **<<0.01** | 2.7 | **<<0.01** | 10.9 | **<<0.01** |

Table S4. The degree of decline of algae/cyanobacteria concentration in the experimental treatments 12 hours after feeding during the equilibrium state of the cultures

|  | Algae supply, mg C L^-1^ | The A experiment ±SD | The B&A experiment ±SD |
| --- | --- | --- | --- |
| *D. longispina* HQ | 0.09 | 2.2-fold | 3.0-fold |
| *D. magna* HQ | 0.09 | 2.9-fold | 3.7-fold |
| *D.longispina &*  *D. magna* HQ | 0.09 | 2.8-fold | 3.3-fold |
| *D. longispina* PP | 0.09 | 1.9-fold | 2.4-fold |
| *D. magna* PP | 0.09 | 2.6-fold | 2.8-fold |
| *D.longispina &*  *D. magna* PP | 0.09 | 2.4-fold | 3.0-fold |
| *D. longispina* CYANO | 0.09 |  | 2.6-fold |
| *D. magna* CYANO | 0.09 |  | 4.1-fold |
| *D.longispina &*  *D. magna* CYANO | 0.09 |  | 4.3-fold |

**List of the abbreviated terms:**
P – phosphorus;

C – carbon;

PUFA – polyunsaturated fatty acids;

EPA – eicosapentaenoic acid;

GLM RM ANOVA – general linear models with repeated-measures ANOVA;

DAPI – 4,6-diamidino-2-phenylindole.

***Types of quality***

HQ – high quality green alga *C. klinobasis* with C:P ≈ 100;

PP – P-poor *C. klinobasis* with C:P ≈ 800;

CYANO – cyanobacteria *S. elongates*.

***Types of experiments***

A = algae/cyanobacteria experiment with small amount of bacteria with changes of medium every day;

B&A = bacteria and algae/cyanobacteria experiment with larger amount of bacteria with changes of medium every other day.
